# A Comparative Study of the Radiological and Functional Outcomes Between the Titanium Elastic Nailing System and Long-Leg Cast Immobilization in Pediatric Femoral Shaft Fractures: A Retrospective Study

**DOI:** 10.7759/cureus.105253

**Published:** 2026-03-15

**Authors:** Amit Kumar Varun, Gils Thampi, Nagakumar J. S.

**Affiliations:** 1 Department of Orthopedics, Sri Devaraj Urs Medical College (SDUMC), Kolar, IND

**Keywords:** limb length discrepancy, long-leg cast, modified rust score, pediatric femoral shaft fracture, titanium elastic nailing

## Abstract

Background: Pediatric femoral shaft fractures are common injuries that can cause significant morbidity and functional impairment if not treated properly. Traditionally, long-leg cast immobilization has been employed; however, prolonged immobilization may lead to joint stiffness, muscle atrophy, limb-length discrepancy, and delayed functional recovery. The titanium elastic nailing system (TENS) provides elastic, stable intramedullary fixation, promotes biological healing, and enables early mobilization. This study compared radiological union, limb length discrepancy, callus formation, pain, range of motion, and complications between TENS fixation and long-leg cast immobilization.

Methods: A retrospective comparative study was conducted involving 60 children aged 5-14 years with diaphyseal femoral fractures treated either with the TENS fixation (n = 30) or long-leg cast immobilization (n = 30). Patients with pathological fractures, open fractures, polytrauma, neuromuscular disorders, or incomplete follow-up were excluded. Follow-up assessments were performed at one, three, and six months, with the final evaluation at six months. Radiological union was assessed using the modified Radiographic Union Scale for Tibial (RUST) fractures score. Limb length discrepancy, callus formation, pain (measured by the Visual Analog Scale), hip and knee range of motion, and complications were also evaluated.

Results: The TENS group demonstrated significantly earlier radiological union (11.2 ± 1.6 vs. 15.8 ± 2.3 weeks; p < 0.001) and higher modified RUST scores at six months. Limb length discrepancy and pain scores were significantly lower in the TENS group. Hip and knee range of motion were superior in the TENS group at the final follow-up. Delayed union occurred in one patient (3.3%) in the TENS group compared with six patients (20%) in the cast group. Cast-related skin complications were observed in five patients (16.7%) in the cast group, while implant irritation occurred in two patients (6.7%) in the TENS group and was managed conservatively.

Conclusion: TENS fixation is an effective treatment option for pediatric femoral shaft fractures, offering earlier radiological union, enhanced functional recovery, and fewer complications related to immobilization than long-leg cast immobilization.

## Introduction

Femoral shaft fractures account for approximately 1%-2% of all pediatric fractures and represent a significant cause of hospitalization and functional disability in children [1]. Traditionally, conservative management involving traction followed by long-leg cast immobilization has been recommended, especially for younger children [2]. However, prolonged immobilization is associated with delayed rehabilitation, joint stiffness, limb length discrepancies, and psychosocial burden [3-5].

With advances in pediatric orthopedics, elastic stable intramedullary nailing using the titanium elastic nailing system (TENS) has become increasingly popular, especially in children aged 5-14 years [6]. TENS provides relative stability, preserves fracture biology, and allows early mobilization [7]. Multiple studies have reported faster union, improved functional recovery, and fewer complications with TENS compared to conservative methods [8-10].

This study aims to compare the radiological and functional outcomes of TENS fixation vs. long-leg cast immobilization in pediatric femoral shaft fractures treated at a rural teaching hospital.

## Materials and methods

This retrospective comparative study was conducted at a tertiary care rural teaching hospital following approval from the Institutional Ethics Committee (SDUAHER/R&D/CEC/SDUMC-PG/314/NF/-2025-26). Medical records of children aged 5-14 years who were treated for isolated femoral shaft fractures between January 2022 and December 2024 were reviewed.

Patients with pathological fractures, open fractures classified as Gustilo-Anderson grade II or higher, associated neurovascular injuries, polytrauma requiring intensive care management, metabolic bone diseases, neuromuscular disorders affecting limb function, previous fractures or surgeries involving the affected femur, segmental fractures, intra-articular fracture extensions, or incomplete clinical or radiological follow-up were excluded. Additionally, patients lost to follow-up before the final six-month assessment were excluded. A total of 60 patients who met the eligibility criteria were included in the study.

Patients were divided into two groups. Group A included 30 patients treated with the TENS fixation, while Group B comprised 30 patients treated with closed reduction and long-leg cast immobilization. Both treatment modalities were routinely practiced during the study period as accepted management options for pediatric femoral shaft fractures. The choice of treatment technique was based on the surgeon's discretion and clinical considerations at the time of management, rather than being determined by the study design. At the time of treatment, no definitive institutional protocol favored one method over the other.

TENS fixation was performed using standard elastic intramedullary nailing techniques under fluoroscopic guidance. Two appropriately sized flexible titanium nails were inserted through medial and lateral entry points at the distal femur and advanced retrograde across the fracture site to achieve stable elastic fixation and maintain proper alignment.

In patients treated with TENS fixation, the operated limb was supported with a temporary splint primarily for comfort and soft-tissue protection for approximately one to two weeks. Prolonged immobilization was not routinely practiced. Early mobilization was initiated once postoperative pain subsided, depending on fracture stability and patient tolerance, thereby providing relative stability and promoting early functional recovery.

All patients were followed up at one, three, and six months (final follow-up). At each visit, a detailed clinical examination and radiological evaluation were performed using standard anteroposterior and lateral radiographs of the affected femur. Fracture healing progression was assessed at one and three months. Definitive radiological union was evaluated at the final six-month follow-up using the modified Radiographic Union Score for Tibial (RUST) fractures scoring system, which assesses cortical bridging and fracture line visibility on follow-up radiographs.

Pain was assessed using the Visual Analog Scale (VAS), and hip and knee range of motion were measured at each follow-up. Limb length discrepancy was evaluated both clinically and radiographically at the final follow-up. Excessive callus formation was defined as a prominent callus exceeding the expected cortical diameter on follow-up radiographs. Complications were documented throughout the follow-up period.

Statistical analysis

Data were analyzed using the Statistical Package for the Social Sciences software, version 26.0 (IBM Corp., Armonk, NY). Continuous variables are presented as means ± standard deviations, while categorical variables are expressed as frequencies and percentages. Intergroup comparisons of continuous variables were performed using independent samples t-tests, and categorical variables were analyzed using the chi-square test. A p value of less than 0.05 was considered statistically significant.

## Results

The mean age of patients in the TENS group was 9.6 ± 2.3 years, which was comparable to 9.9 ± 2.1 years in the cast group (p = 0.62). A similar male predominance was observed in both groups (63.3% vs. 60%; p = 0.79). The distribution of the affected limb showed no significant difference between groups (p = 0.80). Regarding fracture morphology, transverse fractures accounted for 60% of cases in the TENS group and 56.7% in the cast group, while oblique or spiral fractures comprised 40% and 43.3%, respectively (p = 0.79). These findings demonstrate baseline demographic and fracture pattern comparability between the two treatment groups (Table 1).

**Table 1:** Demographic profile of patients at baseline (n = 60) Baseline demographic and fracture characteristics of patients in the TENS and cast groups TENS: titanium elastic nailing system

Parameter	TENS group (n = 30)	Cast group (n = 30)	p value
Mean age (years)	9.6 ± 2.3	9.9 ± 2.1	0.62
Gender (male/female)	19/11	18/12	0.79
Side involved (right/left)	16/14	17/13	0.80
Transverse fractures	18 (60%)	17 (56.7%)	0.79
Oblique/spiral fractures	12 (40%)	13 (43.3%)	0.79

Radiological union occurred significantly earlier in the TENS group, with a mean union time of 11.2 ± 1.6 weeks, compared with 15.8 ± 2.3 weeks in the cast group (p < 0.001). Progressive improvement in cortical healing was observed during serial follow-up assessments. At the final six-month follow-up, the mean modified RUST score was significantly higher in the TENS group (11.6 ± 0.6) than in the cast group (9.2 ± 1.1; p < 0.001), indicating more complete cortical bridging and superior fracture consolidation in patients treated with elastic intramedullary fixation (Table 2).

**Table 2:** Comparison of radiological union and modified RUST score between TENS fixation and long-leg cast immobilization at the final follow-up (six months) (n = 60) TENS: titanium elastic nailing system; RUST: Radiographic Union Scale for Tibial fractures

Parameter	TENS group	Cast group	p value
Time to radiological union (weeks)	11.2 ± 1.6	15.8 ± 2.3	<0.001
Modified RUST score at six months	11.6 ± 0.6	9.2 ± 1.1	<0.001

At the final six-month follow-up, the mean limb length discrepancy was significantly lower in the TENS group (3.4 ± 1.9 mm) compared to the cast group (7.6 ± 3.1 mm; p < 0.001). Excessive callus formation was observed in three patients (10%) in the TENS group, whereas 11 patients (36.7%) in the cast group exhibited excessive callus formation (p = 0.01). These findings indicate improved fracture stability and more controlled biological healing in patients treated with elastic intramedullary fixation (Table 3).

**Table 3:** Comparison of limb length discrepancy and callus formation between TENS fixation and long-leg cast immobilization at the final follow-up (six months) (n = 60) TENS: titanium elastic nailing system

Parameter	TENS group	Cast group	p value
Mean limb length discrepancy (mm)	3.4 ± 1.9	7.6 ± 3.1	<0.001
Excessive callus formation	3 (10%)	11 (36.7%)	0.01

Pain scores were significantly lower in the TENS group at all follow-up intervals. At one month, the mean VAS score was 3.2 ± 0.8 in the TENS group compared to 5.6 ± 1.0 in the cast group (p < 0.001). This difference persisted at three months, with mean scores of 1.9 ± 0.7 and 4.1 ± 1.2, respectively (p < 0.001). At the final six-month follow-up, pain scores remained significantly lower in the TENS group (1.2 ± 0.6) compared to the cast group (3.4 ± 1.1; p < 0.001), indicating sustained pain reduction with elastic intramedullary fixation (Table 4).

**Table 4:** Comparison of pain scores (VAS) at one, three, and six month follow-up (n = 60) TENS: titanium elastic nailing system; VAS: Visual Analog Scale

Parameter	TENS group	Cast group	p value
VAS at 1 month	3.2 ± 0.8	5.6 ± 1.0	<0.001
VAS at 3 months	1.9 ± 0.7	4.1 ± 1.2	<0.001
VAS at 6 months	1.2 ± 0.6	3.4 ± 1.1	<0.001

At the final six-month follow-up, patients treated with TENS fixation achieved near-normal joint mobility. The mean hip range of motion was 96.4% ± 3.1% of normal in the TENS group, compared to 88.2% ± 5.4% in the cast group (p < 0.001). Similarly, knee range of motion was significantly greater in the TENS group (95.1% ± 3.6%) than in the cast group (85.7% ± 6.2%; p < 0.001), indicating superior functional recovery following elastic intramedullary fixation (Table 5).

**Table 5:** Comparison of hip and knee range of motion between TENS fixation and long-leg cast immobilization at the final follow-up (six months) (n = 60) TENS: titanium elastic nailing system; ROM: read-only memory

Parameter	TENS group	Cast group	p value
Hip ROM (% of normal)	96.4 ± 3.1	88.2 ± 5.4	<0.001
Knee ROM (% of normal)	95.1 ± 3.6	85.7 ± 6.2	<0.001

At the six-month follow-up, the overall complication rate was higher in the cast group. Delayed union occurred in six patients (20%) treated with cast immobilization, compared with one patient (3.3%) in the TENS group. Skin-related complications were observed in five patients (16.7%) in the cast group, whereas none were reported in the TENS group. In the TENS group, implant-related irritation due to nail prominence at the entry site was observed in two patients (6.7%) and was managed conservatively without requiring reintervention. Reintervention was necessary in two patients (6.7%) in the cast group. These findings indicate a lower overall complication burden with elastic intramedullary fixation (Table 6).

**Table 6:** Comparison of treatment-related complications between TENS fixation and long-leg cast immobilization at the final follow-up (six months) (n = 60) TENS: titanium elastic nailing system

Complication	TENS group (n = 30)	Cast group (n = 30)
Delayed union	1 (3.3%)	6 (20%)
Skin complications	0	5 (16.7%)
Implant irritation	2 (6.7%)	0
Reintervention	0	2 (6.7%)

## Discussion

The present study demonstrates that fixation using the TENS results in superior radiological and functional outcomes compared with long-leg cast immobilization in pediatric femoral shaft fractures. The earlier fracture union observed in the TENS group aligns with previously published literature, highlighting the biological and mechanical advantages of elastic stable intramedullary fixation [8-12]. The shorter mean time to radiological union in the TENS group may be attributed to stable internal fixation, preservation of the periosteal blood supply, and controlled micromotion at the fracture site, all of which promote callus formation and early cortical bridging.

Higher modified RUST scores at the six-month follow-up in the TENS group indicate more reliable cortical healing compared to conservative management. These findings align with studies by Ligier et al. and Flynn et al., who demonstrated predictable fracture union and early mobilization using titanium elastic nails [6,12]. The elastic stability provided by TENS allows physiological load sharing across the fracture site while maintaining proper alignment, thereby enhancing fracture healing and reducing the risk of delayed union.

Improved fracture stability achieved with TENS fixation also resulted in significantly lower limb length discrepancy and reduced excessive callus formation. Limb length discrepancy following femoral shaft fractures in children is a well-recognized complication, particularly with conservative treatment, due to fracture instability and stimulation of the growth plates [13-15]. The lower incidence of limb length discrepancy observed in the TENS group in the present study supports the role of internal fixation in maintaining fracture alignment and minimizing postfracture overgrowth.

Pain reduction and improved hip and knee range of motion observed in the TENS group can be attributed to early mobilization, shorter immobilization periods, and the avoidance of prolonged casting. Early mobilization helps prevent joint stiffness, muscle atrophy, and functional decline, which are commonly associated with long-leg cast immobilization. Similar functional advantages of TENS fixation, including enhanced joint mobility and an earlier return to normal activities, have been documented in multiple comparative studies [16-19]. These functional benefits are especially important in the pediatric population, where prolonged immobilization may negatively impact both physical and psychological well-being.

The higher complication rate observed in the cast group, particularly delayed union and skin-related issues, further underscores the limitations of conservative management. Prolonged immobilization increases the risk of pressure sores, skin breakdown, and challenges in maintaining cast hygiene, especially in younger children. In contrast, complications associated with TENS fixation in the present study were minimal and manageable, with no cases requiring reintervention, supporting the safety and reliability of this technique when appropriately indicated.

However, certain practical considerations must be acknowledged when interpreting these findings. TENS fixation typically requires a planned second procedure for implant removal after fracture healing, which may involve additional anesthesia exposure and increased healthcare costs. Furthermore, the biomechanical stability of elastic intramedullary fixation depends on selecting an appropriate nail diameter relative to the medullary canal and the patient's body habitus. In heavier or older adolescents, inadequate nail-to-canal diameter ratios may predispose patients to complications such as angulation or telescoping. Therefore, careful patient selection and strict adherence to standard technical principles are essential for achieving optimal outcomes.

Overall, the findings of this study support the growing body of evidence favoring the TENS as an effective and reliable treatment modality for pediatric femoral shaft fractures, particularly in school-aged children.

This study also has several strengths. It offers a direct comparative evaluation of operative versus conservative management of pediatric femoral shaft fractures within a single institutional setting, utilizing standardized follow-up intervals and consistent outcome measures. The inclusion of both radiological and functional outcome parameters enables a comprehensive assessment of fracture healing and postoperative recovery.

Limitations

This study has several limitations. Its retrospective design may introduce selection bias and rely on the accuracy of medical records. The relatively small sample size may limit the generalizability of the findings. Additionally, the six-month follow-up period may be insufficient for a definitive assessment of limb length discrepancy, as post-traumatic femoral overgrowth in children can continue for 18-24 months after fracture healing. Furthermore, TENS fixation requires a planned second procedure for implant removal, which entails additional exposure to anesthesia and costs compared with conservative treatment. Patient weight and body mass index were not separately analyzed in this study, although standard intraoperative principles for nail selection based on canal diameter were followed. Finally, the study's single-center design may limit its external validity.

## Conclusions

TENS fixation is a safe and effective treatment modality for pediatric femoral shaft fractures. Compared with long-leg cast immobilization, TENS provides faster radiological union, better preservation of limb length, superior pain control, improved hip and knee joint mobility, and a lower complication rate. These advantages facilitate earlier rehabilitation and functional recovery, making TENS the preferred treatment option for appropriately selected pediatric patients.

## References

[1] Kocher MS, Sink EL, Blasier RD (2009). Treatment of pediatric diaphyseal femur fractures. J Am Acad Orthop Surg.

[2] Shapiro F (1981). Fractures of the femoral shaft in children: the overgrowth phenomenon. Acta Orthop Scand.

[3] Reeves RB, Ballard RI, Hughes JL (1990). Internal fixation versus traction and casting of adolescent femoral shaft fractures. J Pediatr Orthop.

[4] Flynn JM, Hresko T, Reynolds RA, Blasier RD, Davidson R, Kasser J (2001). Titanium elastic nails for pediatric femur fractures: a multicenter study of early results with analysis of complications. J Pediatr Orthop.

[5] Canale ST, Tolo VT (1995). Fractures of the femur in children. Instr Course Lect.

[6] Ligier JN, Metaizeau JP, Prévot J, Lascombes P (1988). Elastic stable intramedullary nailing of femoral shaft fractures in children. J Bone Joint Surg Br.

[7] Metaizeau JP (2004). Stable elastic intramedullary nailing for fractures of the femur in children. J Bone Joint Surg Br.

[8] Heinrich SD, Drvaric DM, Darr K, MacEwen GD (1994). The operative stabilization of pediatric diaphyseal femur fractures with flexible intramedullary nails: a prospective analysis. J Pediatr Orthop.

[9] Luhmann SJ, Schootman M, Schoenecker PL, Dobbs MB, Gordon JE (2003). Complications of titanium elastic nails for pediatric femoral shaft fractures. J Pediatr Orthop.

[10] Narayanan UG, Hyman JE, Wainwright AM, Rang M, Alman BA (2004). Complications of elastic stable intramedullary nail fixation of pediatric femoral fractures, and how to avoid them. J Pediatr Orthop.

[11] Sink EL, Gralla J, Repine M (2005). Complications of pediatric femur fractures treated with titanium elastic nails: a comparison of fracture types. J Pediatr Orthop.

[12] Flynn JM, Skaggs DL, Sponseller PD (2002). The operative management of pediatric fractures of the lower extremity. J Bone Joint Surg Am.

[13] Canavese F, Marengo L, Andreacchio A (2016). Complications of elastic stable intramedullary nailing of femoral shaft fractures in children weighing fifty kilograms (one hundred and ten pounds) and more. Int Orthop.

[14] Wallace ME, Hoffman EB (1992). Remodelling of angular deformity after femoral shaft fractures in children. J Bone Joint Surg Br.

[15] Li H, Liu J, Wang D (2024). Origin and factors for overgrowth in pediatric fractures of the femoral shaft after flexible intramedullary nail fixation. Transl Pediatr.

[16] Wright JG, Wang EE, Owen JL (2005). Treatments for paediatric femoral fractures: a randomised trial. J Bone Joint Surg Br.

[17] Caird MS, Mueller KA, Puryear A, Farley FA (2003). Compression plating of pediatric femoral shaft fractures. J Pediatr Orthop.

[18] Buechsenschuetz KE, Mehlman CT, Shaw KJ, Crawford AH, Immerman EB (2002). Femoral shaft fractures in children: traction and casting versus elastic stable intramedullary nailing. J Trauma.

[19] Sanders JO, Browne RH, Mooney JF (2001). Treatment of femoral fractures in children by pediatric orthopedists: results of a 1998 survey. J Pediatr Orthop.

